# Retraction: Downregulation of microRNA-218 is cardioprotective against cardiac fibrosis and cardiac function impairment in myocardial infarction by binding to MITF

**DOI:** 10.18632/aging.203548

**Published:** 2021-09-14

**Authors:** Linfeng Qian, Shaobo Pan, Liping Shi, Yongyi Zhou, Lai Sun, Zhedong Wan, Yufang Ding, Jia Qian

**Affiliations:** 1 Department of Cardiothoracic Surgery, First Affiliated Hospital of Zhejiang University, Hangzhou, 310003, PR. China; 2 Operating Room, First Affiliated Hospital of Zhejiang University, Hangzhou, 310003, PR. China

**Keywords:** retraction

Original article: Aging. 2019; 11:5368–5388. . https://doi.org/10.18632/aging.102112

**This article has been retracted:** The authors recently found that the images in Figures 2, 3, 7, 8, and 10 contain errors that cannot be resolved. The authors now feel that the experimental results are not solid enough to support their conclusions. They have therefore made the decision to retract the article with the agreement of all listed authors.

The Administration of the First Affiliated Hospital of Zhejiang University was notified about the retraction by Aging Journal.

